# FRAILTY AND CARDIOVASCULAR DISEASE EVENTS IN COMMUNITY-DWELLING HEALTHY OLDER ADULTS

**DOI:** 10.1093/geroni/igac059.1301

**Published:** 2022-12-20

**Authors:** A R M Saifuddin Ekram, Sara Espinoza, Joanne Ryan, Michael Ernst, Andrew Tonkin, Christopher Reid, John McNeil, Robyn Woods

**Affiliations:** Monash University, Nunawading, Victoria, Australia; Sam and Ann Barshop Institute, UT Health San Antonio, San Antonio, Texas, United States; Monash University, Melbourne, Victoria, Australia; The University of Iowa, Iowa City, Iowa, United States; Monash University, Melbourne, Victoria, Australia; Curtin University, Perth, Western Australia, Australia; Monash University, Melbourne, Victoria, Australia; Monash University, Melbourne, Victoria, Australia

## Abstract

**BACKGROUND:**

Frailty is associated with adverse outcomes, but whether it independently increases cardiovascular disease (CVD) risk requires clarification.

**METHODS:**

This study examined the association between frailty in a cohort with no previous CVD events and subsequent CVD outcomes in 19,114 community-dwelling older people from the ASPREE trial. Frailty was assessed using the modified Fried phenotype, comprising weakness, exhaustion, low body mass index (BMI), slowness and low physical activity, and a deficit accumulation frailty index (FI) of 66 items. CVD event was defined as a composite of CVD death, non-fatal myocardial infarction, non-fatal stroke and hospitalization for heart failure.

**Results:**

Over a median 4.7-years of follow-up (interquartile range: 3.6 to 5.7 years), pre-frail/frail participants were more likely to develop CVD events (Hazard ratio (HR): 1.33; 95% Confidence Interval (CI): 1.16, 1.53 for pre-frail and HR: 1.68; 95% CI: 1.19, 2.38 for frail participants) according to Fried phenotype. Subtypes of CVD (fatal/non-fatal myocardial infarction and heart failure hospitalization) similarly increased HRs except fatal or non-fatal stroke. These effect sizes were more prominent when frailty was assessed using the FI than that assessed by Fried phenotype.

**CONCLUSION:**

Pre-frail and frail participants were at significantly increased risk of developing CVD and its sub-types (particularly fatal/non-fatal myocardial infarction and hospitalization for heart failure). Addressing pre-frailty and frailty in older people could contribute to CVD prevention strategies.

